# Bilateral Serous Retinal Detachment and Posterior Reversible Encephalopathy Syndrome Precipitated by Eclamptic Attack

**DOI:** 10.7759/cureus.13444

**Published:** 2021-02-19

**Authors:** Rahaf A Mandura

**Affiliations:** 1 Department of Ophthalmology, King Abdulaziz University, Jeddah, SAU

**Keywords:** serous retinal detachment, eclampsia, posterior reversible encephalopathy syndrome

## Abstract

Eclampsia is a severe hypertensive disease accompanied by tonic-clonic convulsions in the second half of pregnancy or during delivery in the absence of other aetiology. The association between eclampsia and serous retinal detachment is not common. We report a case of a 20-year-old primipara with eclampsia who developed bilateral exudative retinal detachment after delivery. Brain computed tomography (CT) reported findings in the occipital lobes consistent with posterior reversible encephalopathy syndrome. A few weeks after delivery, there was spontaneous resorption of subretinal fluid and complete resolution of bilateral serous retinal detachment with residual signs of choroidal ischemia, including Elschnig’s spot, Siegrist streak and some pigmentary changes in the retinal pigment epithelium. Visual acuity returned to normal in both eyes and there was complete resolution of the disease.

## Introduction

Pre-eclampsia is a hypertensive disease that includes high blood pressure (>140/90 mmHg) in combination with proteinuria (>300 mmHg), pretibial oedema or both [1]. It occurs in the second half of pregnancy or during delivery in the absence of other aetiologies [2-4]. Eclampsia is the most severe form of this disease with mostly systolic pressure to be found >200 mmHg, oliguria or anuria, and in most severe cases tonic-clonic convulsions occur [5].

Posterior reversible encephalopathy syndrome (PRES), which is also known as reversible posterior leukoencephalopathy syndrome (RPLS) was first described as a single named syndrome in a case series by Hinchey in 1996 [6]. It was defined as a reversible syndrome manifested as headache, confusion or decreased level of consciousness, seizures, and loss of vision associated with neuroimaging findings suggestive of posterior cerebral white matter oedema [7,8].

Ophthalmic complications have been documented in 30% to 100% of patients with pre-eclampsia [2]. Serous retinal detachment (SRD) is a rare ocular manifestation first described by Von Graefe in 1885 [2]. It has been documented to occur in 1% to 2% of patients with severe pre-eclampsia and 10% of patients with eclampsia [2,8]. However, to the best of our knowledge, the simultaneous occurrence of PRES and SRD in pre-eclampsia/eclampsia is an extremely rare condition based on the literature review.

## Case presentation

**Figure 1:**
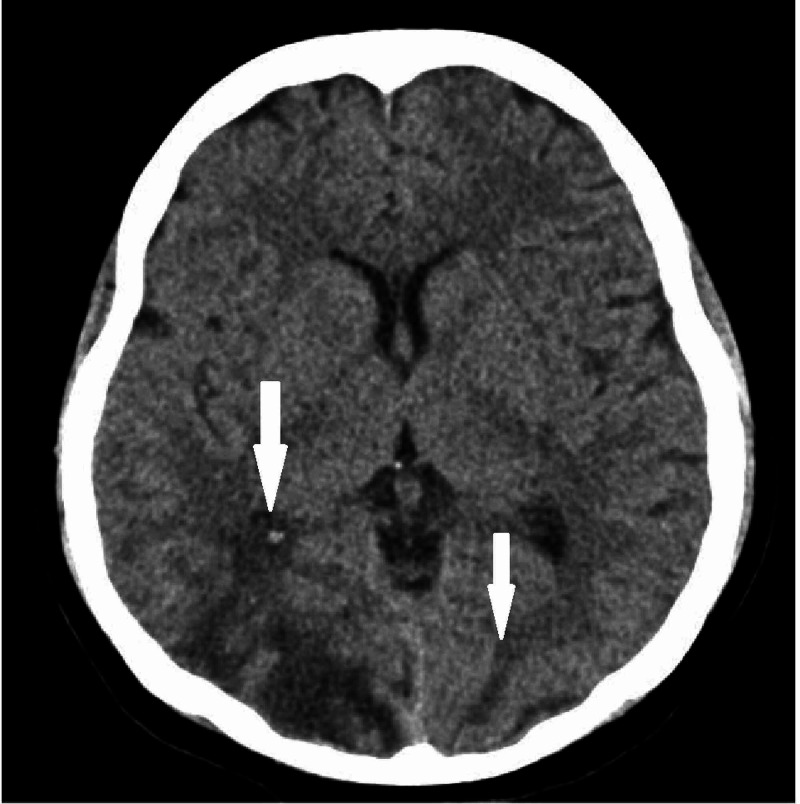
Computed tomography (CT) scan of the brain showed bilateral asymmetrical cortical and subcortical hypodense areas at bilateral occipital lobes (arrows), a finding consistent with posterior reversible encephalopathy syndrome (PRES).

A 20-year-old primipara was referred from the obstetrics ward to the ophthalmology department at King Abdulaziz University Hospital for evaluation of bilateral visual loss. She was admitted through the emergency room as a case of eclampsia. Upon arrival at the emergency department (ED), she was confused with a blood pressure of 230/137 mmHg. Her accompanying relative reported the occurrence of one seizure episode before arriving at the hospital. The physical examination further revealed generalized body oedema and bilateral pretibial pitting oedema, while her laboratory analysis showed the presence of proteinuria +3. Obstetric ultrasound revealed oligohydramnios, and the non-stress test revealed signs of intrauterine compromise. These findings seemed to favour a primary diagnosis of pre-eclampsia or eclampsia. However, there were no signs of the patient developing haemolysis elevated liver enzymes low platelet levels syndrome (HELLP syndrome). Regarding her laboratory workup, haemoglobin was 14 g/dL, alanine aminotransferase 58 U/L, aspartate aminotransferase 72 U/L, alkaline phosphatase 355 U/L, gamaglutamyle transferase 32 U/L, total bilirubin 4 µmol/L, lactate dehydrogenase 551 U/L, and platelet count of 125 K/µL. Furthermore, her bleeding profile showed elevated fibrinogen level of 554 mg/dL, prothrombin time 10 seconds, partial prothrombin time 30.5 seconds, and INR 0.9. Neurological evaluation and computed tomography (CT) scan of the brain showed bilateral asymmetrical occipital hypodensity, a finding consistent with PRES (Figure 1).

An emergency caesarean section was performed on the same day. Shortly after delivery, she reported to have severe occipital headache, dizziness, and total loss of vision of both eyes with a blood pressure of 197/130 mmHg. Intravenous labutolol (Trandate) and hydralazine (Apresoline) were administered again to control he blood pressure and was reduced to 165/100 mmHg. An elevated D-dimer level of 11.3 mg/L and fibrinogen 554.2 mg/dL raised the suspicion of cerebral venous sinus thrombosis, which was subsequently ruled out by brain CT venography. In the subsequent postpartum days, blood pressure was controlled at a level between 140 and 150/90 mmHg with oral nifedipine (Adalat) 40 mg thrice daily and oral methyldopa (Aldomet) 250 mg, given twice a day in which the laboratory parameters of the patient started to normalize down the line.

**Figure 2:**
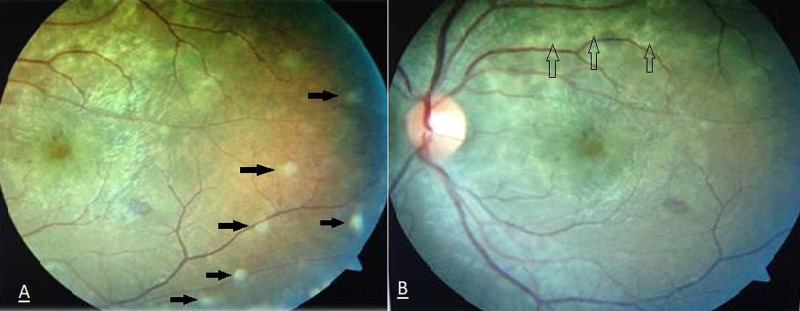
Fundus examination showed signs of choroidal ischemia including (A) Elschnig’s spot and (B) Siegrist streak and resolved serous retinal detachment.

On the second postpartum day, the patient was referred to the ophthalmology department to investigate her vision loss. On examination, visual acuity was 20/200 in both eyes. On slit-lamp examination, bilateral SRDs involving the macula were seen in both eyes with no retinal tears or breaks. Optic discs were normal bilaterally and the anterior chambers were clear with no cells noted in the eyes. The initial eye examination was done at the bedside due to the patient’s medical condition. Therefore, we could not perform fundus photo or optic coherence tomography (OCT) at the given time. A conservative management approach was undertaken with watchful waiting under close observation for spontaneous resolution of SRD. During the follow-up visit a week later, her vision had dramatically improved. The visual acuity of the right eye was 20/30 and that of the left eye was 20/40. Intraocular pressure (IOP) measured with an air buff tonometer revealed an IOP of 7 mmHg in the right eye and 12 mmHg in the left eye. Fundus examination showed signs of choroidal ischemia, including Elschnig’s spot and Siegrist streak, and the SRD was improved (Figure 2).

**Figure 3:**
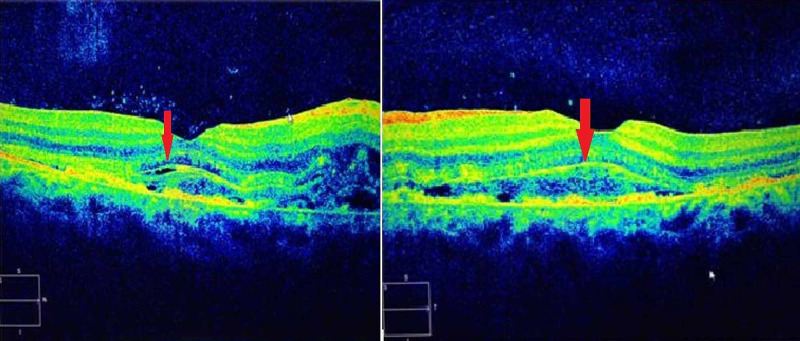
Optic coherence tomography of the macula showing subretinal fluid involving fovea in both eyes.

OCT model 2000 (Zeiss-Humphrey Instrument Inc., San Leandro, CA, USA) with axial and longitudinal resolution of 10-20 µm was used for macular mapping and showed subretinal fluid, which involved the macula and fovea in both eyes (Figure 3).

**Figure 4:**
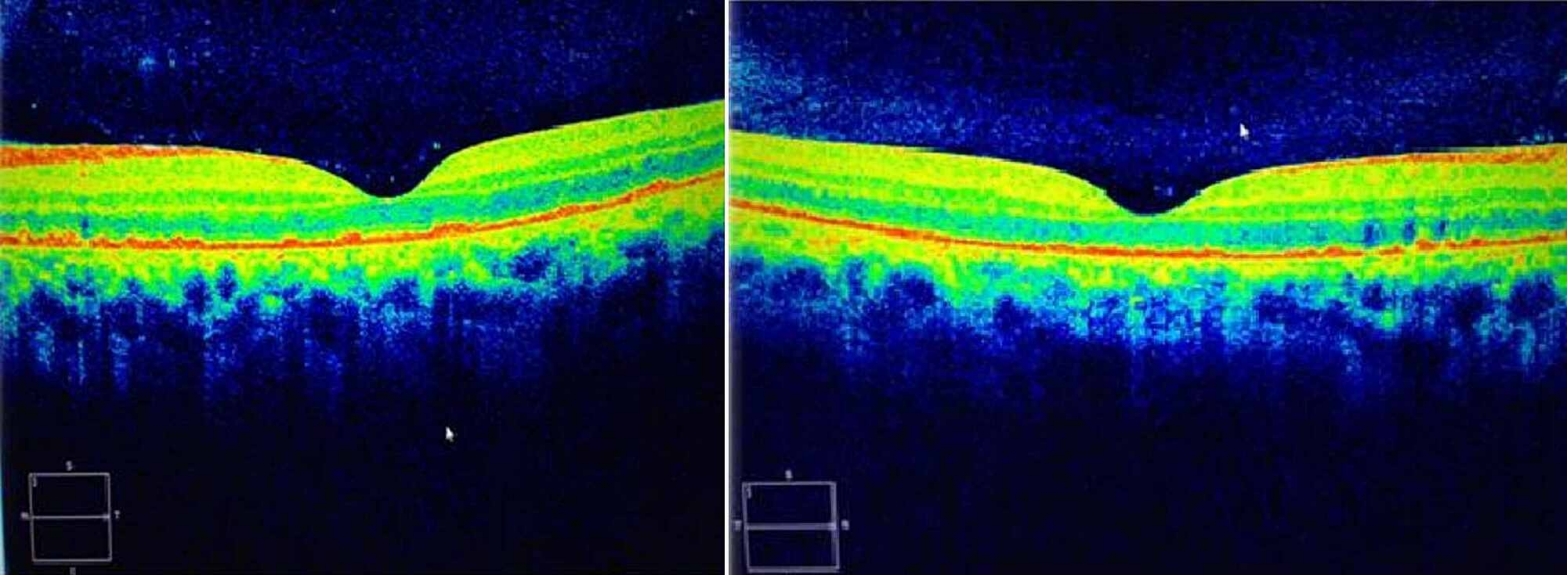
Optic coherence tomography of the macula showing complete resolution of subretinal fluid in both eyes.

In the next follow-up visit two weeks' postpartum, visual acuity was 20/25 on the right eye and 20/40 on the left eye and subretinal fluid has resolved spontaneously. After one month, the patient's vision nearly normalized to 20/25 bilaterally and the macular oedema resolved completely in macular OCT (Figure 4).

## Discussion

The pathogenesis of retinal detachment in pre-eclampsia is not well understood. However, it has been attributed to damage in choroidal vasculature and subsequent ischemia, which is triggered by endogenous choriocapillaris vasoconstrictor chemicals [9,10]. Breaking the outer blood-retinal barrier leads to exudation into subretinal space, causing SRD [9]. The mainstay of management of pre-eclampsia is early detection, control of blood pressure, and delivery before irreversible complication occurs [11]. Ophthalmic management of pre-eclampsia-associated SRD is conservative and spontaneous resolution generally occurs in few weeks with good visual recovery to pre-detachment level [12]. The visual outcome is less favourable in the presence of retinopathy features, including haemorrhage, exudates, and cotton-wool spots [13].

The pathogenesis of PRES is quite unknown yet [10]. In the literature, there are two proposed theories to explain pathogeneses. The first one is the failure of cerebral vascular autoregulation in response to systemic hypertension, mainly in the vertebrobasilar circulation, which results in the breakdown of the blood-brain barrier and vasogenic oedema [10]. The other theory proposes a much similar mechanism to that of SRD, which involves endothelial dysfunction, which induces both capillary leakage and vasoconstriction, leading to ischemia and subsequent oedema [6,7,10]. Our case report supports the observation of the common mechanism of endothelial dysfunction in SRD and PRES, which causes oedema both across the retinal pigment epithelium and in the brain [10].

## Conclusions

In conclusion, we emphasize the fact that different factors play a role in the visual impairment of patients with eclampsia and support the importance of the identification of the possible association between PRES and exudative retinal detachment in these patients which is crucial for proper management. Bilateral SRD and PRES caused by pre-eclampsia can be managed conservatively. However, close observation is required to avoid further complications.
